# Bacterial screening of platelet donations in England, 2014–2023

**DOI:** 10.1111/vox.70253

**Published:** 2026-03-23

**Authors:** Vidushi Chugh, Shannah Secret, Katy Davison, Autumn St John, Peter Simmonds, Susan R. Brailsford, Heli Harvala

**Affiliations:** ^1^ Radcliffe Department of Medicine University of Oxford Oxford UK; ^2^ Microbiology Services NHS Blood and Transplant London UK; ^3^ NHSBT and UK Health Security Agency (UKHSA) Epidemiology Unit UKHSA London UK; ^4^ Institute of Biomedicine, Faculty of Medicine University of Turku and Turku University Hospital Turku Finland; ^5^ Nuffield Department of Medicine University of Oxford Oxford UK

**Keywords:** apheresis, bacterial contamination, near‐misses, platelets, pooled, transfusion‐transmitted infections

## Abstract

**Background and Objectives:**

Bacterial contamination of blood components is an ongoing problem in transfusion medicine. We analysed the bacterial screening data of platelets from England, 2014–2023, and compared this with data on reported near‐misses and transfusion‐transmitted infections (TTIs).

**Materials and Methods:**

Anonymized data on bacterial screening of pooled and apheresis platelet donations were reviewed, including the number of donations collected yearly, results from bacterial screening and time from sampling to detection. The findings were compared with data on near‐misses and TTIs reported during the same period.

**Results:**

Screening of 1249,513 apheresis and 1,495,707 pooled platelet donations identified bacterial contamination in 2949 donations, including 78 bacterial species. Over four‐fold higher frequency of confirmed bacterial contamination was observed in pooled platelets compared to apheresis donations (0.09% [1096/1,249,513] vs. 0.02% [362/1,495,707], *p* < 0.0001). Rates of bacterial contamination of pooled platelet doubled during the study period. *Staphylococcus aureus* was the most commonly detected highly pathogenic bacterial contaminant (29/147, 19.7%; 15/29, 52% in apheresis platelets). It was also implicated in 1 confirmed case of bacterial TTI and in 8 of 10 reported bacterial near‐miss cases.

**Conclusion:**

Increasing frequencies of bacterial contamination, mostly related to skin flora, were noted in pooled platelets. Furthermore, *S. aureus* was notably associated with near‐miss events. Our findings demonstrate a limitation of bacterial screening, with evidence of bacterial growth after platelets were likely supplied for clinical use.

**Highlights**

- High pathogenic bacterial contamination was detected more often in pooled platelets than in apheresis platelets.
- Almost half of initial bacterial‐positive cultures were confirmed (1451/3010, 48%), indicating that the associated platelet donation was received back at the blood services for testing and not used clinically.
- The other half of initial positive cultures (1559/3010, 52%) could not be investigated further to confirm or exclude contamination because they were either transfused or discarded, and hence the results were labelled indeterminate.

## INTRODUCTION

Historically, bacterial contamination of platelet components constitutes the greatest microbiological safety risk to transfusion recipients, as bacteria can continue to multiply during storage at 22°C before transfusion. Steps taken to reduce the risk of bacterial contamination include guidelines for donor selection [1], diversion of the initially collected blood and optimized donor arm disinfection. Application of pathogen reduction [2] and bacterial screening are further key measures that reduce the risk of bacterial transmission through transfusion. Bacterial screening was adopted as a safety measure in many blood transfusion services globally following the recommendation from the World Health Organization [3]. The methods employed for bacterial screening are usually culture‐based but can also rely on large‐volume delayed sampling and, perhaps in the future, on universal detection of conserved 16S ribosomal RNA (rRNA) of bacteria combined with next‐generation sequencing methods [4].

In England, bacterial screening of all platelet components was introduced in 2011 by NHS Blood and Transplant (NHSBT) following a pilot study. Automated bacterial screening has since been used to screen all buffy coat pooled platelets and apheresis platelet donations. Both anaerobic and aerobic blood culture bottles are inoculated with an 8‐mL sample taken from the platelet unit no earlier than 36 h after the blood donation, with 6 h post‐incubation hold, and incubated up to 7 days in total. This holding period enables bacteria to proliferate to a sufficient number in a platelet donation to be detected in the test sample, yielding a calculated sensitivity of 1–10 CFU/mL [5]. Platelets are released as ‘negative to date’ if no bacterial growth is detected within the first 6 h of testing in the bacterial culture system. Prior to the implementation of bacterial screening, 40 bacterial transfusion‐transmitted infections (TTIs) were reported in England and 11 of those were fatal. In contrast, only a single confirmed bacterial TTI involving *Staphylococcus aureus* has been reported since [6, 7].

This study aims to evaluate the effectiveness and limitations of bacterial screening of platelet donations in England between 2014 and 2023. Specifically, we have analysed the number and types of bacteria detected over time in apheresis and pooled platelet donations and compared those with the number of reported near‐misses and bacterial TTIs. Additionally, we have analysed the time from collection to detection of different bacterial species and used that data to discuss the effectiveness and limitations of the current bacterial screening strategy.

## MATERIALS AND METHODS

### Data collection

Anonymized data on bacterial screening of platelets in England between 2014 and 2023 were provided by the NHSBT/UK Health Security Agency (UKHSA) Epidemiology team. For each apheresis donation (derived from a single donor) and pooled platelet donation (consisting of recovered platelets from four donors), the dataset included the donation type, donation date, bacterial screening result, identified bacterial species (if any) and the time interval from sampling to detection.

### Bacterial screening of platelets

Apheresis donations were collected using the Trima Accel automated blood collection system by Terumo, whereas pooled donation manufacturing switched from the Optipress II (Baxter, United States) to an LMB device (Lmb Technologie GmbH, Germany) in 2018, as detailed earlier [8].

Bacterial screening was performed as previously reported [1, 4]. Inoculated aerobic and anaerobic culture bottles were incubated in the BacT/ALERT 3D automated microbial detection system (bioMérieux UK Ltd., Basingstoke, UK). Units associated with initially reactive cultures were subsequently referred for confirmatory testing, which was performed in duplicate when associated platelet packs were available. Bacterial screening results are reported as negative (no growth in bacterial culture) or initially reactive (reactivity detected in bacterial culture bottle). Initially reactive were subsequently reported as confirmed positive (growth of the same bacterial species from the initial screening bottle and re‐culturing of index platelet unit and any associated blood pack), indeterminate positive (positive results re‐generated either in bacterial culture bottle or the blood pack itself but not both), indeterminate negative (no growth of re‐culturing the initially reactive blood culture bottle, and associated units were not available for culture) or confirmed negative (no growth detected in the subsequent testing of the culture bottle or index platelet unit).

In data analysis, we have further divided bacterial species into three groups based on their likely pathogenicity: high‐, moderate‐ and low‐pathogenicity strains. High‐pathogenicity bacterial species (modified from reference [8] to include *Campylobacter*, *Enterobacter*, *Enterococcus*, *Klebsiella oxytoca*, *Proteus mirabilis* and *Staphylococcus lugdunensis* due to their clinical relevance) are bacteria with the potential to cause clinical harm (moderate) and bacteria that are usually skin‐commensal and rarely cause any clinical infections (low pathogenicity). For each bacterium, we have analysed the time taken from sampling to detection.

For indeterminate positives associated with apheresis platelets in 2023, we identified whether the hospital had been informed of these findings and whether the unit was issued and used or discarded either at NHSBT or at the hospital.

### Bacterial TTIs and near‐miss events

Data on TTIs and near‐miss events reported to the UK haemovigilance scheme, Serious Hazards of Transfusion (SHOT), between 2014 and 2023 were analysed. In addition, data on TTIs were also collated from 2000 to 2010 to reflect the time period before the bacterial screening was implemented. In near‐miss events, the platelet donation pack was identified as visually abnormal and not transfused. These donations were investigated further for bacterial growth.

SHOT is a passive haemovigilance system that relies on hospital transfusion staff to note and report visually abnormal packs to enable recall of associated packs as soon as possible. It also advises patients to report to the hospital staff any symptoms that occur within 24 h of transfusion [9] and then report these promptly to SHOT and the blood services to allow recall and further investigations [10]. It is to be noted that acute transfusion reactions can vary in their severity from minor febrile reactions to life‐threatening allergic, haemolytic or hypotensive events. However, hospitals are informed and advised to recall all bacterially contaminated platelet units they have received and reminded to investigate any possible bacterial TTI; they will contact NHSBT if they have concerns in relation to this.

A TTI is confirmed where a recipient acquires infection post a blood transfusion. This is noted in the case where there is no evidence of a prior infection and it is established that a blood component from the same donor was the source of the infection or that the pathogen was present in the component.

### Statistical analysis

Differences in the proportion contaminated between either pooled and apheresis and different time periods 2014–2018 and 2019–2023 were compared using Chi‐square test within Vassar Stats (Statistical Computation Web Site, http://www.vassarstats.net/), with *p*‐values ≤0.05 considered statistically significant. Trendlines were investigated using least squares regression, and *R* ^2^ was calculated to assess model fit.

## RESULTS

**FIGURE 1:**
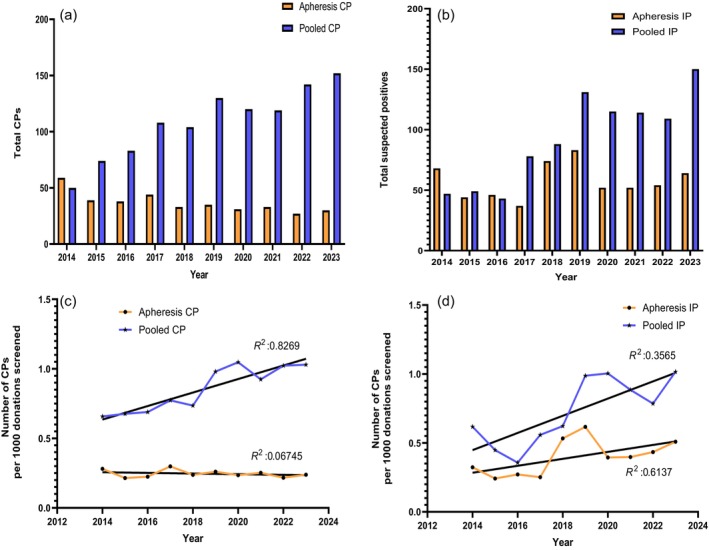
Total number of confirmed positives (CPs) (a) pooled and apheresis donations and (b) indeterminate positives (IPs) for pooled and apheresis donations in bacterial screening from 2014 to 2023. Prevalence of CP (c) and IP (d) apheresis and pooled donations calculated per 1000 donations from 2014 to 2023.

During this 10‐year study, a total of 2,745,220 platelet components, comprising 1,249,513 (45.4%) pooled and 1,495,707 (54.5%) apheresis platelet donations, were collected and subjected to bacterial screening by NHSBT. From these, 5833 (0.21%) donations were initially reactive in a bacterial culture bottle. A higher number of initial reactives were observed for pooled platelets than for apheresis platelets donations (3232, 0.25% from 1,249,513 vs. 2601, 0.17% from 1,495,707, respectively) (Figure 1). Of these, 1451 donations were confirmed positive for bacterial contamination with over four‐fold higher percentages observed in pooled platelets than apheresis donations (0.09% [1096/1,249,513] vs. 0.02% [362/1,495,707], *p* < 0.0001), respectively (Figure 1). Of the remaining initially reactive donations, 1559 were reported as indeterminate positive (965 pooled and 594 apheresis donations), indicating that the components were not returned to NHSBT and hence contamination could not be confirmed or excluded.

The number of confirmed bacterial contaminations of pooled platelet donations increased by 58% over the study period from 419 (from 586,947 screened) in 2014–2018 to 663 (from 662,566 screened) in 2019–2023, whereas the number of bacterially contaminated apheresis donations declined by 27% from 213 (from 848,104 screened) in 2014–2018 to 156 (from 647,603 screened) in 2019–2023 (Figure 1). There was a similar increase in platelet units with indeterminate contamination in both pooled and apheresis donations (Figure 1): from 305 in 2014–2018 to 619 in 2018–2023 for pooled platelets and from 269 to 305 for apheresis donations. Although this coincided with a gradual increase in the number of pooled platelet donations manufactured in England (from 76,094 in 2014 to 147,630 in 2023), the rate of confirmed and indeterminate bacterial contamination in pooled platelets increased from 2014–2018 to 2019–2023 (0.52–0.93 per 1000 and 0.71–1.00 per 1000 donations, respectively, Chi‐square test for both changes, *p* < 0.0001, Figure 1). In contrast, the frequency of confirmed and indeterminate bacterial contamination in apheresis platelets has remained relatively unchanged.

**FIGURE 2:**
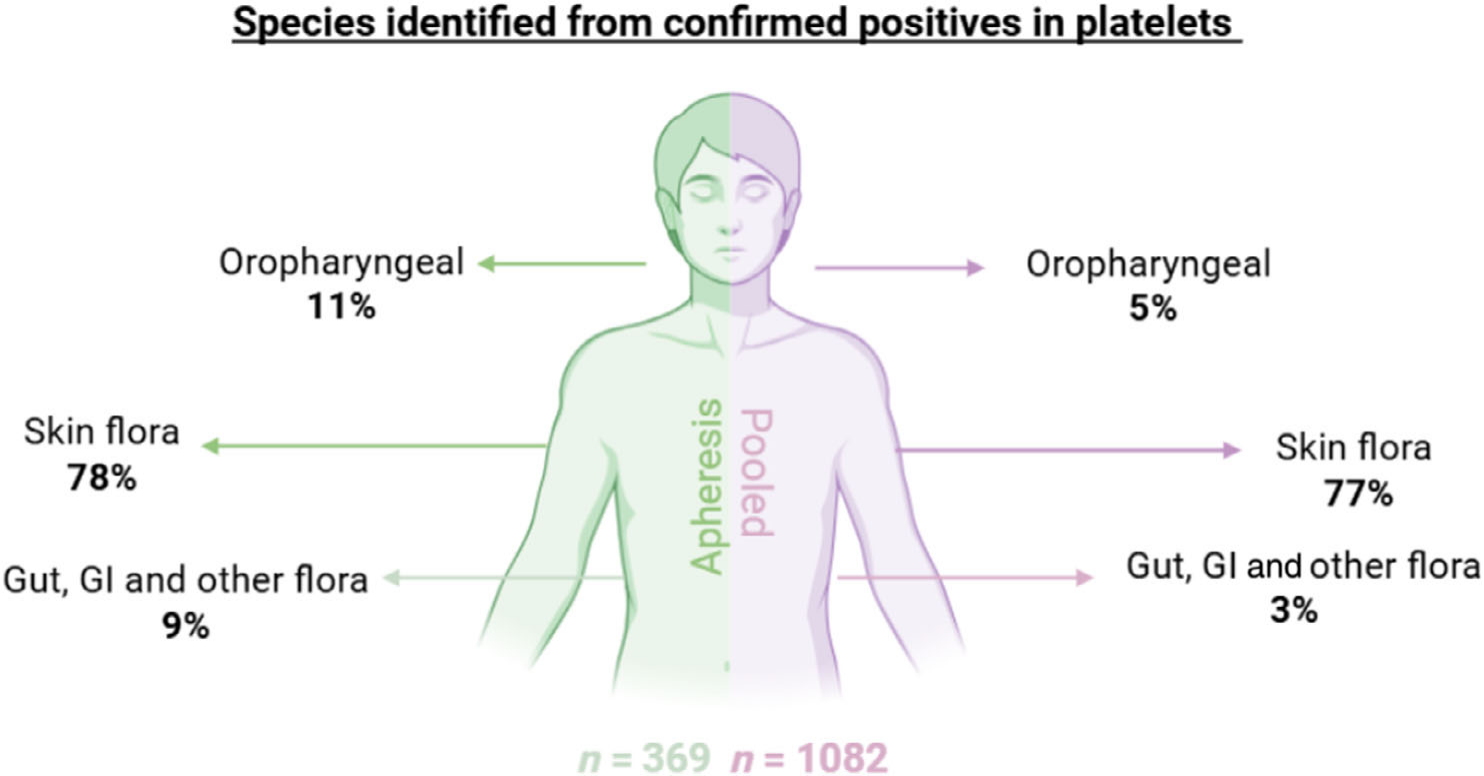
Anatomical site of bacteria isolated from 2014 to 2023 in apheresis and pooled donations in positives only. GI, gastrointestinal.

A total of 78 bacterial species were identified among 2949 apheresis and pooled platelet donations classified as confirmed or indeterminate positive following bacterial screening, and from these, 23 species accounted for indeterminate positives. In healthy blood donors, the source of bacterial contamination is considered to be endogenous and, in most cases, originates from a person's own commensal microbial flora. *Cutibacterium* spp. and other skin flora comprised a greater proportion of confirmed and indeterminate positives in pooled than apheresis platelets (1454/1650, 89% vs. 692/854, 81%, *p* < 0.001), whereas gut and oropharyngeal flora were more common in apheresis platelet donations as opposed to pooled platelets (34/854, 5% vs. 44/1650, 3%), which is not considered highly pathogenic for gut flora and (82/854, 10% vs. 77/1650, 5%, *p* < 0.0001) for oropharyngeal flora (all by Chi‐square test; Figure 2).

**FIGURE 3:**
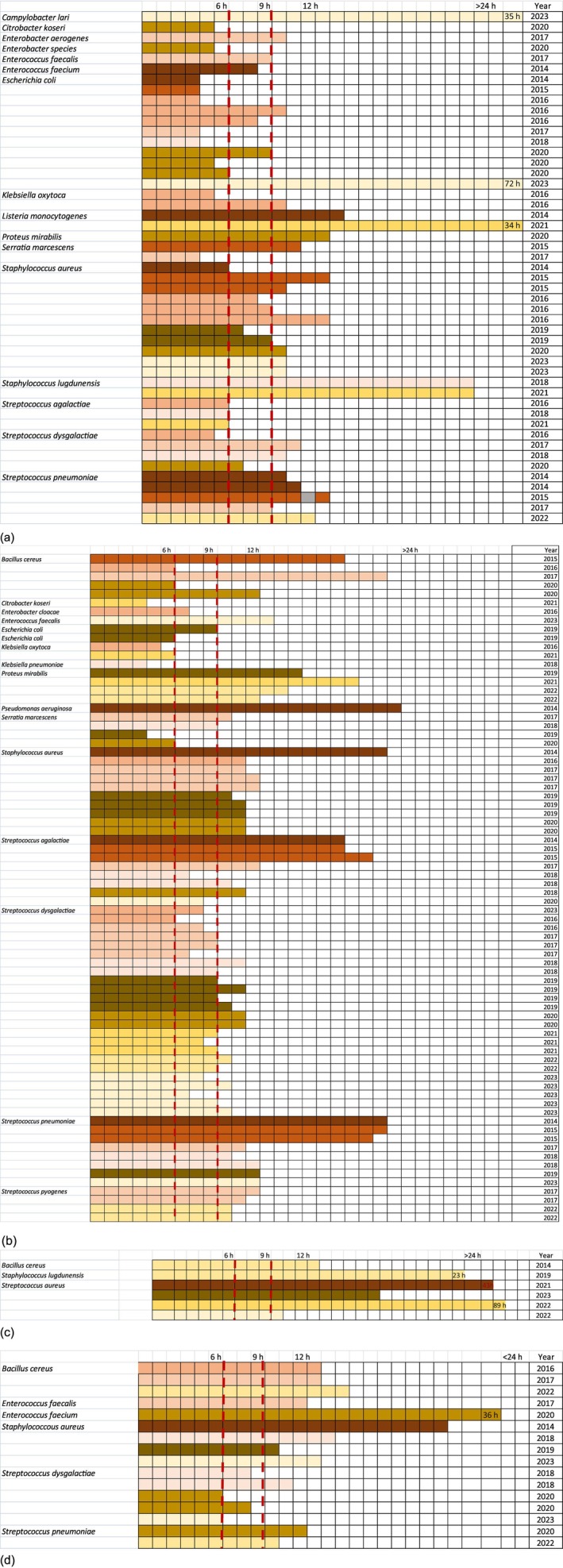
Time to detection of the initial reactive screen bottles with highly pathogenic bacterial species, January 2014 to December 2023, England. Data for confirmed positive apheresis (a, *n* = 52) and pooled platelet donations (b, *n* = 73, data missing for 3), and for indeterminate positive apheresis (c, *n* = 6) and pooled platelet donations (d, *n* = 17). The dashed lines for 6 and 9 h, respectively, represent the current post‐incubation hold at NHS Blood and Transplant and the earliest time point when the donation would be most likely supplied to clinical use if the bacterial culture remained negative. (a) Apheresis platelet donations with high pathogenic bacteria detected (*n* = 52). (b) Pooled platelet donations with high pathogenic bacteria detected (*n* = 75; 3 data missing). (c) Apheresis platelet donations with indeterminate bacteria of high pathogenicity detected (*n* = 6). (d) Pooled platelet donations with indeterminate bacteria of high pathogenicity detected (*n* = 17).

Eighteen different bacterial species isolated from confirmed and indeterminate positive units were considered highly pathogenic (Figure 3; 147 isolates, 125 of these confirmed positives). Over 60% of these were detected in pooled platelet donations (76/125 of confirmed positives and 16/22 of indeterminate positives). Highly pathogenic bacterial contaminants that were confirmed positive in apheresis and pooled platelet donations included *S. aureus* (21/125, 17%, respectively) likely originating from the skin, *Streptococcus agalactiae* (9%, 11/125), *Escherichia coli* (10%, 12/125), *Serratia marcescens* (5%, 6/125) and *Citrobacter koseri* (1.6%, 2/125) originating from the gut, *Streptococcus pneumoniae* (10%, 13/125), *Streptococcus dysgalactiae* (28/125, 22%), *Streptococcus pyogenes* (3%, 4/125) and *Klebsiella pneumoniae* (0.8%, 1/125) of oropharyngeal origin and other bacteria without clear endogenous source, including *Listeria monocytogenes* (1.6%, 2/125), *Bacillus cereus* (4%, 5/125) and *Pseudomonas aeruginosa* (0.8%, 1/125).

Seven different bacterial species isolated from indeterminate positive units were considered highly pathogenic (Figure 3c,d; 22 isolates). The indeterminate bacterial contaminants of apheresis and pooled platelet donations likely originated from the skin included *S. aureus* and *S. lugdunensis* (8/22, 36% and 1/22, 4.5%); those of oropharyngeal origin included *S. dysgalactiae*, *S. pneumoniae* and *Enterococcus faecalis* (5/22, 23%; 2/22, 9% and 1/22, 4.5%) and bacteria without a clear endogenous source included *B. cereus* (4/22, 18%). Furthermore, 442 isolates from 23 species were classified as moderate pathogenicity and 1916 isolates from 18 species as low pathogenicity (Tables S1 and S2; total of 2358 isolates).

Even though the total number of bacterially contaminated platelets increased over the study period (2014–2023), there was no increase in the number of highly pathogenic strains detected in either donor groups. There was also no significant change in the number of skin contaminants of high pathogenicity over time; indeed, only around 1 in 100,000 platelet donations collected were found to harbour skin contaminants.

The bacterial detection times for confirmed positives varied largely, with a median detection time of 56 h for apheresis donations (average 60 h, range 1–143 h) compared to 74 h for pooled platelet donations (average 67 h, range 0–149 h) (Figure 3). Only 6% of these confirmed bacterial contaminations were detected before 6 h with ‘negative to date’ results, noting that confirmatory testing could easily be done as the donations had not left NHSBT yet. Furthermore, the detection times for confirmed positives with high pathogenicity varied from 3.9 h (*S. marcescens*) to 24 h (*P. aeruginosa*) for pooled platelets and from 3.7 to 72 h (*E. coli*) for apheresis platelets. Only a small proportion of highly pathogenic strains were detected in pooled or apheresis platelet donations by 6 h (8/66, 12% and 14/41, 34%, respectively). Furthermore, for indeterminate positives, around 10% of suspected bacteria were detected before the release at 6 h (2/25). The detection times for indeterminate positives with high pathogenicity varied from 6 h (*E. coli*) to 89 h (*S. aureus*) for apheresis platelets, and from 6 to 36 h for pooled platelets (*S. aureus*).

The follow‐up data on 64 indeterminate positives associated with apheresis platelet donations in 2023 showed that hospitals were notified and advised to recall all platelet units contaminated with any bacteria. The platelet unit contaminated with high‐pathogenicity bacteria was successfully recalled, whereas two apheresis packs contaminated with moderate‐ and 31 of those with low‐pathogenicity bacteria had already been transfused, noting all except 1 of these were slow‐growing *Cutibacterium* spp. (Table 1). The average detection time for high‐ and moderate‐pathogenicity bacteria was shorter than that for the low‐pathogenicity ones (11, 34 and 92 h, respectively).

**TABLE 1:** Bacterial contamination in apheresis donations (indeterminate positives) recalled in 2023 by pathogenicity and range.

Bacterial species	Total identified	Pathogenicity	Range or reactive time (h)	Total donations	Total packs from donations	Discarded/issued
*Staphylococcus aureus*	1	High	10.5	1	1	1 discarded
*Staphylococcus epidermis*	14	Moderate	19–78	14	15	14 discarded; 1 issued
*Streptococcus mitis/oralis*	2	Moderate	21.7	2	2	2 discarded
Coagulase negative staph	1	Moderate	14–36	1	1	1 discarded
*Bacillus* species	1	Moderate	21.2	1	2	1 discarded; 1 issued
*Staphylococcus saprophyticus*	1	Moderate	26.5	1	1	Discarded
*Clostridium* species	1	Moderate	19	1	1	1 discarded
*Streptococcus* species	3	Moderate	11–59	3	3	3 discarded
Gram positive bacteria	1	Moderate	27	1	1	1 discarded
*Actinomyces oris/vicsosus*	2	Low	36–79	2	2	2 discarded
*Cutibacterium* species	36	Low	4–131	36	46	16 discarded; 30 issued
*Microbacterium arborescens*	1	Low	60	1	1	1 issued
Total	64	‐	‐	64	76	43 discarded; 33 issued

Between 2014 and 2023, 991 suspected bacterial incidents were reported and investigated by NHSBT (Table 2). From these, one confirmed bacterial TTI linked to *S. aureus* was reported and one possible case linked to *Staphylococcus capitis*. In comparison, 26 confirmed bacterial TTIs were reported in England between 2000 and 2010 before bacterial screening was implemented (Table 3). These included 15 different bacterial species, from which 3, namely *B. cereus*, *K. pneumoniae* and *Pseudomonas koreensis*, were linked to fatalities. Most reported transmissions were linked to pooled platelets (*n* = 15), followed by seven transmissions via apheresis platelets and four via red blood cell transfusions.

**TABLE 2:** Number of near‐misses related to platelet donations reported in England, 2014–2023.

Year	Number of suspected bacterial incidents reported and investigated	Number of post‐transfusion reactions with no evidence of bacteria in investigations	Not TTI	Number of near‐misses	Number of confirmed TTIs	Number of possible TTIs
2014	39	1	36	2^x^	0	0
2015	24	3	19	0	1	1
2016	108	90	14	4^3x1y^	0	0
2017	106	96	9	0	0	1
2018	97	80	15	1^x (late detection)^	0	1
2019	139	130	9	0	0	0
2020	135	122	12	1^x^	0	0
2021	115	110	5	0	0	0
2022	115	62	52	1^x^	0	0
2023	113	97	16	0	0	0
Total	991	791	187	9	1	1

*Note*: All except detections in 2018 and 2020 were in apheresis platelets. ^x^ *Staphylococcus aureus* detected *n* = 7. ^y^ *Serratia marcescens* detected *n* = 1.

Abbreviation: TTIs, transfusion‐transmitted infections.

**TABLE 3:** Confirmed bacterial transfusion‐transmitted infections identified in England before the implementation of bacterial screening, 2000–2010 (*n* = 26).

	Number of cases
Source component	
Pooled platelets	15
Apheresis platelets	7
Bacterial species implicated	
*Staphylococcus epidermis*	6
*Staphylococcus aureus*	2
*Streptococcus pneumoniae*	2
Group B *Streptococcus*	3
*Streptococcus bovis*	1
*Bacillus cereus*	1
*Klebsiella pneumoniae*	3
*Escherichia coli*	1
*Enterobacter cloacae*	1
*Morganella morganus*	1
Associated fatalities	
*B. cereus* from platelets	1
*K. pneumoniae* from platelets	2

Nine platelet donations that had been provided for clinical use as culture‐negative on screening were classed as near‐misses after hospital staff had noted and reported visible clumping of material in the blood donation packs before transfusions, between 2014 and 2023. These included six donations contaminated with *S. aureus*, one of which was classified as late detection, and one with *Serratia* spp. (Table 2). The incidents involved apheresis platelets in three cases and pooled platelets in four cases; all *S. aureus* contaminations were in apheresis donations, and all were negative after 7 days of bacterial culture.

## DISCUSSION

Bacterial screening of 2.7 million platelet donations in England during a 10‐year period between 2014 and 2023 identified almost 6000 initially reactive platelet donations, and around half of these were confirmed as positive or indeterminate positive by further testing. Over 70% of these positives were detected in pooled platelets (1822/2589) with a gradual increase in the detections from 2014 to 2023. Although this partially reflects the increase in pooled platelet collections, there has also been a two‐fold increase in the rate of confirmed and indeterminate bacterial contamination in pooled platelets—from 97 in 2014 to 302 in 2023.

Increasing collections of pooled platelets is seen as a potentially cheaper alternative to implementing further apheresis collection facilities in response to the increasing demand for platelets. The risk associated with the transfusion of pooled platelets has been widely discussed. It was initially debated whether the recipients of pooled platelets would be at four‐fold higher risk of acquiring TTIs due to pooling, leading to the increased donor exposures than those receiving apheresis‐derived platelets [11, 12], or whether the risk would be only two‐fold higher when other donor exposures relating to additional blood components are taken into consideration [13, 14]. However, based on our data, pooled platelet donations were four times more likely to be contaminated with bacteria than apheresis platelets throughout the study period. Although only a fraction of these positives were linked to bacterial species with high pathogenicity, the low‐pathogenicity strains cannot be simply discounted as clinically non‐significant. The clinical significance of contamination is dependent on the dose of bacteria transfused, the bacterial strain and the susceptibility of the patient, variables not captured in this analysis. This potential risk associated with the increasing use of pooled platelets must be acknowledged, and the artificial classification of bacterial contaminants into high, moderate and low pathogenicity must also be interpreted with caution, as there is no acceptable level or species of bacterial contamination [15].

*Cutibacterium* spp. and other skin flora accounted over 80% of contaminants detected in pooled platelets. Where arm cleansing and discard of the first 30 mL of collected blood was introduced to minimize the likelihood of skin flora contamination during the collection process, it is difficult to scientifically prove where this contamination occurs. The manufacturing process of pooled platelets, usually utilizing closed systems but involving handling of tubing and containers to combine products, might increase the risk of exposure of the component to bacterial contamination [16]. *Cutibacterium* spp. has also been linked to bacterial flora of periodontitis, underscoring its dual role as a skin‐ and oral‐associated contaminant (31% in periodontitis donors vs. 10% in periodontally healthy donors) [17]. Other studies have also reported higher contamination rates for pooled platelets than for apheresis platelets (0.604% vs. 0.156%), most likely originating from the skin flora [12, 15]. Furthermore, over 50% of all reported bacterial TTIs reported in England between 2000 and 2010 were associated with pooled platelets (Table 3).

Nine bacterial near‐miss incidents were noted from 2014 to 2023, seven caused by *S. aureus*. These were all missed by bacterial screening of platelets but detected on visual inspection before the transfusion was initiated. Furthermore, one confirmed TTI linked to *S. aureus* was reported in England in 2015. This highlights the utility and importance of visual inspection of platelet components prior to release, as it is known that automated bacterial culture cannot fully eliminate the risk of bacterial contamination [18, 19]. The variability in detection by bacterial screening is likely due to the complicated growth characteristics of *S. aureus*. It has been speculated that this could be related to biofilm formation: fibrinogen‐binding and membrane surface proteins produced by *S. aureus* bind to receptors on platelets, causing their aggregation, and while hampering their detection, making them more visible to the eye. Alternative explanations include the presence of bacteriophages that may help the bacterium to evade detection through inhibiting their replication and reducing the bacterial load [20]. Future research into *S. aureus*‐associated bacteriophages could help clarify whether phage‐mediated lysis or genetic variability contributes to missed detections of *S. aureus* in blood donation screening, ultimately improving diagnostic accuracy and blood safety.

Despite the late detection of many bacterial contaminants in platelet donations, our data provide reassurance that at least half were detected before the units were transfused. Firstly, almost half of the indeterminate positives were confirmed (1451/3010, 48%), indicating that the associated platelet donation was received for testing by the blood services. Secondly, despite a total of 991 suspected bacterial incidents reported and investigated by blood services over the 10‐year study period, only 1 confirmed and 1 possible bacterial TTI were identified. However, the biggest limitation of our system is the lack of data connection from the blood services to the hospital (vein‐to‐vein), which would help monitor the fate of the platelet donations shown to be contaminated with bacteria and facilitate subsequent recipient investigations. Furthermore, passive haemovigilance reporting relies on awareness of clinical members of transfusion teams to consider the possibility of any post‐transfusion reactions, noting septic transfusion reactions are frequently unrecognized or unreported [21]. This challenge is further compounded by the frequent use of antibiotic therapy in recipients, which may suppress bacterial detection and prevent recognition of the bacterial TTI [22]. It is also important to note that bacteria may not survive in the donation packs because of the complex growth characteristics unique to each bacterial species [23].

Bacterial contamination in platelet donations often shows delayed detection, which can then lead to near‐misses and decreased blood safety. Molecular techniques such as 16S rRNA PCR followed by nanopore sequencing might be able to resolve this issue provided the required sensitivity is achieved. This technique could furthermore provide additional data on genotypes and possible co‐infections [24]. While molecular tests will be able to detect any residual nucleic acids, additional transcriptomic analysis and more sophisticated bioinformatic tools are required to classify whether those reads relate to a true infection or originate from non‐viable organisms and contaminants [25]. 16S rRNA PCR had been previously piloted for platelet screening in Germany and Brazil but not implemented into routine national screening because of its suboptimal sensitivity of around 10 CFU/mL [26, 27, 28]. If the sensitivity could be improved, the use of molecular methods for bacterial screening immediately prior to clinical release could circumvent the need for complex recall procedures, including the associated red blood cell units, and ensure greater confidence in blood safety. Furthermore, other methods such as enhanced large‐volume delayed sampling with 48 h pre‐sampling hold and 12 h post‐incubation should be investigated, as they have been shown to improve the sensitivity of bacterial screening by 50% in Canada and the United States in comparison to 36 h pre‐sampling hold and 6 h post‐incubation applied by NHSBT in England [29, 30]. Whether it could improve safety without compromising supply should be investigated further, also in England.

During this 10‐year study period, bacterial screening of 2,745,220 platelet components identified 2949 donations with bacterial contamination, and only 1 confirmed bacterial TTI was reported. Despite this, platelet donations collected in England demonstrated a substantial increase in the rate of bacterial contaminations, although the detected bacteria were mostly considered clinically non‐significant. It will be important to identify how these contaminations occurred and could be prevented. Our findings also clearly demonstrate a limitation of bacterial screening: namely bacterial growth often becomes detectable only after the platelet donation had potentially been supplied for clinical use. Whether the extended large‐volume delayed sampling or the molecular screening utilizing 16S rRNA PCR without compromising the sensitivity of screening could address this limitation remains to be seen.

## CONFLICT OF INTEREST STATEMENT

The authors declare no conflicts of interest.

## Supporting information


**Table S1.** Moderate pathogenic bacterial species isolated from screened apheresis and pooled platelet components: time to detection of the initial‐reactive screen bottles, January 2014 to December 2023, England. Data include both confirmed and indeterminate positives.
**Table S2.** Minimally pathogenic bacterial species isolated from screened apheresis and pooled platelet components: time to detection of the initial‐reactive screen bottles, January 2014 to December 2023, England. Data include both confirmed and indeterminate positives.

## Data Availability

The data that support the findings of this study are available from the corresponding author upon reasonable request.
