# Supplementary material for: Bacterial screening of platelet donations in England, 2014–2023
Source: Vox Sang. 2026 Mar 23;121(7):999–1009. doi: 10.1111/vox.70253 (PMC13356991; doi:10.1111/vox.70253)
Supplement: Supplementary file 1 — Table S1. Moderate pathogenic bacterial species isolated from screened apheresis and pooled platelet components: time to detection of the initial‐reactive screen bottles, January 2014 to December 2023, England. Data include both confirmed and indeterminate positives. Table S2. Minimally pathogenic bacterial species isolated from screened apheresis and pooled platelet components: time to detection of the initial‐reactive screen bottles, January 2014 to December 2023, England. Data include both confirmed and indeterminate positives. [file VOX-121-999-s001.docx]

**Supplementary Table 1.** Moderate pathogenic bacterial species isolated from screened apheresis and pooled platelet components: time to detection of the initial-reactive screen bottles, January 2014 to December 2023, England. This data includes both confirmed and indeterminate positives.

|  | |  | | | | Type of Donation | | | | | | | | | | | |  |  |
| --- | --- | --- | --- | --- | --- | --- | --- | --- | --- | --- | --- | --- | --- | --- | --- | --- | --- | --- | --- |
|  |  | Both | |  | | Apheresis | | | |  | | | Pooled | | | | |  | |
| Bacterial Species | Detected >6hr | | | | | Total isolated | | | Time (hr) |  | | Total Isolated | | | | Time (hr) | |  |  |
| Skin commensals | |  | | | |  | |  | | | |  | | |  | | |  |  |
| *Staphylococcus saprophyticus* | | | Y | | 1 | | 27 | | | | 2 | | | 2 | | |  |  |  |
| *Staphylococcus epidermis* | | | Y | | 49 | | 14-120 | | | | 107 | | | 2-83 | | |  |  |  |
| *Staphylococcus hominis* | | | Y | | 5 | | 14-121 | | | | 13 | | | 2-105 | | |  |  |  |
| *Pseudomonas stutzeri* | | | Y | |  | |  | | | | 1 | | | 25 | | |  |  |  |
| *Streptococcus mitis/oralis* | | | Y | | 71 | | 7-43 | | | | 6 | | | 14-29 | | |  |  |  |
| *Corynebacterium spp.* | | Y | | | | 8 | | 23-119 | | | | 13 | | | 21-101 | | |  |  |
| Skin flora/eczema | | Y | | | |  | |  | | | |  | | |  | | |  |  |
| *Coagulase-Negative Staphylococci* | | Y | | | | 10 | | 4-61 | | | | 20 | | | 4-61 | | |  |  |
| *Finegoldia magna* | | Y | | | | 2 | | 42-71 | | | |  | | |  | | |  |  |
| *Streptococcus anginosus* | | Y | | | | 5 | | 26-59 | | | |  | | |  | | |  |  |
| Orpharyngeal bacteria | |  | | | |  | |  | | | |  | | |  | | |  |  |
| *Abiotrophia spp.* | | Y | | | | 1 | | 24 | | | | 1 | | | 34 | | |  |  |
| *Aggregatibacter aphrophilus* | | Y | | | | 2 | | 33-41 | | | |  | | |  | | |  |  |
| *Parvimonas micra* | | Y | | | | 3 | | 55-93 | | | | 2 | | | 56-145 | | |  |  |
| *Slackia exigua* | | N | | | | 1 | | 74 | | | |  | | |  | | |  |  |
| *Streptococcus spp.* | | Y | | | | 41 | | 5-98 | | | | 20 | | | 6-61 | | |  |  |
| Gut bacteria | |  | | | |  | |  | | | |  | | |  | | |  |  |
| *Clostridium species* | | Y | | | | 1 | | 19 | | | |  | | |  | | |  |  |
| *Peptostreptococcus spp.* | | Y | | | | 5 | | 45-98 | | | | 2 | | | 41-61 | | |  |  |
| *Parabacteroides distasonis* | | Y | | | |  | |  | | | | 1 | | | 42.5 | | |  |  |
| *Klebsiella aerogenes* | | Y | | | | 1 | | 10 | | | |  | | |  | | |  |  |
| Water bacteria | |  | | | |  | |  | | | |  | | |  | | |  |  |
| *Aeromonas species* | | Y | | | |  | |  | | | | 1 | | | 10 | | |  |  |
| *Bacilus spp.* | | Y | | | | 15 | | 19-105 | | | | 24 | | | 4-140 | | |  |  |
| *Paenibacillus spp.* | | Y | | | | 2 | | 31-46 | | | | 4 | | | 33-81 | | |  |  |
| *Pantoea agglomerans* | | Y | | | |  | |  | | | | 1 | | | 18-27 | | |  |  |
| *Stenotrophomonas maltophilia* | | Y | | | | 1 | | 38 | | | |  | | |  | | |  |  |
| Total | |  | | | | **224** | |  | | | | **218** | | |  | | |  |  |

**Supplementary Table 2.** Minimally pathogenic bacterial species isolated from screened apheresis and pooled platelet components: time to detection of the initial-reactive screen bottles, January 2014 to December 2023, England. This data includes both confirmed and indeterminate positives.

|  | | | Type of Donation | | | | | | | | | | | | | | | | |  |
| --- | --- | --- | --- | --- | --- | --- | --- | --- | --- | --- | --- | --- | --- | --- | --- | --- | --- | --- | --- | --- |
|  |  | |  | Both |  | | Apheresis | | | |  | | | | Pooled | | | | |  |
| Bacterial Species |  | |  | Detected >6 hrs |  | | Total isolated | |  | Time (hr) |  | | | Total isolated | | | |  | Time (hr) |  |
| Skin commensals | | |  | | | |  | |  | | | |  | | | |  | | |  |
| *Cutibacterium spp.* | | Y | | | | 534 | | 4-143 | | | | 1297 | | | | 1-148 | | | |  |
| *Dermabacteraceae.* | | Y | | | |  | |  | | | | 1 | | | | 65 | | | |  |
| *Kocuria spp.* | | Y | | | | 1 | | 66 | | | | 5 | | | | 29-128 | | | |  |
| *Microbacterium arborescens* | | Y | | | | 1 | | 60 | | | |  | | | |  | | | |  |
| *Micrococcus species* | | Y | | | | 5 | | 46-128 | | | | 19 | | | | 12-125 | | | |  |
| Orpharyngeal bacteria | |  | | | |  | |  | | | |  | | | |  | | | |  |
| *Acinetobacter lwoffii* | | Y | | | |  | |  | | | | 1 | | | | 73 | | | |  |
| *Actinomyces spp.* | | Y | | | | 7 | | 20-66 | | | | 2 | | | | 20-63 | | | |  |
| *Fusobacterium nucleatum* | | Y | | | | 3 | | 51-59 | | | | 1 | | | | 49 | | | |  |
| *Fusobacterium varium* | | Y | | | |  | |  | | | | 1 | | | | 22 | | | |  |
| *Granulicatella adiacens* | | Y | | | | 3 | | 15-19 | | | |  | | | |  | | | |  |
| *Haemophilus aphrophilus* | | Y | | | | 1 | | 56 | | | |  | | | |  | | | |  |
| *Lancefieldella parvula* | | Y | | | | 1 | | 61 | | | |  | | | |  | | | |  |
| *Lactobacillus spp.* | | Y | | | | 2 | | 29-48 | | | |  | | | |  | | | |  |
| Gut bacteria | |  | | | |  | |  | | | |  | | | |  | | | |  |
| *Bifidobacterium spp.* | | Y | | | | 4 | | 65-86 | | | |  | | | |  | | | |  |
| *Bacteroides spp.* | | Y | | | | 4 | | 100-122 | | | | 11 | | | | 20-143 | | | |  |
| *Collinsella aerofaciens* | | Y | | | |  | |  | | | | 1 | | | | 46 | | | |  |
| *Gemella spp.* | | Y | | | | 8 | | 11-90 | | | | 2 | | | | 2-23 | | | |  |
| Water bacteria | | Y | | | |  | |  | | | |  | | | |  | | | |  |
| *Caldibacillus thermoamylovorans* | | Y | | | | 1 | | 36 | | | |  | | | |  | | | |  |
| Total | |  | | | | **575** | |  | | | | **1341** | | | |  | | | |  |
